# LIM-domain binding protein 2 was down-regulated by miRNA-96-5p inhibited the proliferation, invasion and metastasis of lung cancer H1299 cells

**DOI:** 10.1016/j.clinsp.2022.100145

**Published:** 2022-12-05

**Authors:** Fuying Chu, Xinxin Xu, Yan Zhang, Hua Cai, Jingjing Peng, Yanan Li, Han Zhang, Hongli Liu, Xiang Chen

**Affiliations:** a Department of Laboratory Medicine, Nantong First People's Hospital, China; b Department of Laboratory Medicine, Affiliated Hospital of Nantong University, China; c Department of Laboratory Medicine, Nantong Tumor Hospital, China

**Keywords:** LDB2, miR-96-5p, Proliferation, Invasion, Metastasis, Lung cancer

## Abstract

**Objectives:**

Lung cancer was one of the most common malignancies around the world. It has great significance in to search for the mechanism of occurrence and development of lung cancer. LIM Domain Binding protein 2 (LDB2) belongs to the LIM-domain binding family, it can be used as a binding protein that combined with other transcription factors to form the transcription complex for regulating the expression of target genes. The expression of microRNA-96-5p (miR-96-5p) has been investigated in various tumors. The aim of this study is to investigate the potential role of LDB2 and miR-96-5p in lung cancer.

**Methods:**

Real-time quantitative PCR was applied to detect the expression of LDB2 and miR-96-5p. The proliferation, invasion, and metastasis of H1299 cells were analyzed by CCK8, transwell, and wound healing assay after LDB2 or miR-96-5p transfection. Luciferase activities assay and western blot were used to reveal the targeted regulation between LDB2 and miR-96-5p.

**Results:**

Here the authors found LDB2 was down-regulated in lung cancer tissues and negatively correlated with miR-96-5p expression, it could promote or inhibit the proliferation, invasion and metastasis of H1299 cells after LDB2 knockdown or overexpression and regulate the expression of cyclinD1, MMP9, Bcl-2, and Bax via ERK1/2 signaling pathway. Furthermore, miR-96-5p exerted its function by directly binding to 3′-UTR of LDB2 and regulating expression of LDB2. miR-96-5p could promote the proliferation, invasion, and metastasis of H1299 cells.

**Conclusion:**

These findings demonstrate that LDB2 can act as a new regulator to inhibit cell proliferation, invasion, and metastasis via the ERK1/2 signaling pathway, and miR-96-5p may be a potential promising molecular by targeting LDB2 in lung cancer.

## Highlights

• This is the first time to verify the targeting regulation of miR-96-5p on LDB2.
• MiR-96-5p/LDB2 regulates cellular behaviors through ERK1/2 signaling pathway in lung cancer H1299 cell.

## Introduction

Lung cancer is the most common cancer with high morbidity and mortality in the world. According to histology classification, it includes Small Cell Lung Cancer (SCLC) and Non-Small Cell Lung Cancer (NSCLC). Particularly, NSCLC accounts for more than 85% of lung cancer cases, and the 5-year survival rate of patients remains poor.^1^ In recent years, with the development of diagnostic and therapeutic techniques, and the introduction of small molecule targeted drugs and immunotherapy, the effect of treatment in lung cancer has been improved remarkably. However, the overall prognosis of patients remains poor. Therefore, the authors need to research the molecular mechanisms and search the potential diagnostic markers and therapeutic targets for lung cancer.

LDB2, also known as CLIM-1, belongs to the Lim domain binding family and functions as a transcriptional regulatory factor, is widely expressed in a variety of human tissues.^2^ The LDB family is composed of three members, LDB1, LDB2 and LDB3, which are highly conserved in two homologous domains: an amino-terminal homodimerization Domain (DD) and a carboxyl-terminal LIM Interaction Domain (LID).^3^ LDB proteins can form multimeric complexes through DD and mediate long-range promoter-enhancer to form a chromatin loop. LDB proteins lack DNA binding capacity and are thought to be unable to directly be involved in transcriptional regulation. However, by interacting with multiple LIM-Homeodomain (LIMHD) and LIM-Only (LMO) proteins through their LID, LDBs can well be able to regulate transcription. Through these domains, LDB proteins can bring together multiple interacting proteins into high-order large multiprotein complexes that are involved in multiple developmental pathways.^4^ LDB1, the homologous protein of LDB2, has been widely explored such as erythroid differentiation, embryogenesis, and cancer development,^5^, ^6^, ^7^ but the biological role of LDB2 bearing 78% identity and 89% similarity to the LDB1 is largely unknown. Recently, the role of LDB2 in cancer has attracted people's attention, but its role in tumors is still controversial. Yu et al.^8^ had demonstrated LDB2 could inhibit proliferation and migration in liver cancer cells. Overexpression of LDB2 remarkably weakened the influence of tumor suppressor factor ERRAC on the viability of CRC cells.^9^ Lately, Zhai et al.^10^ reported that LDB2 was involved in regulating the proliferation of lung cancer cells, but the function of LDB2 in other biologies such as invasion and metastasis needs to be further studied.

MicroRNAs (miRNAs) are endogenous and non-coding single-stranded microRNAs with about 21-23nt in length. MiRNAs can inhibit mRNA translation or promote mRNA degradation by binding to their 3′-Untranslated Region (3′-UTR), and thus inhibit gene expression at the post-transcriptional level.^11^ As we all know, hundreds of distinct miRNAs have been found so far. Many studies have shown that miRNAs were abnormally expressed in lung cancer. For example, miR-519d-3p suppressed tumorigenicity and metastasis by inhibiting Bcl-w and HIF-1α in NSCLC.^12^ MiRNA-199 expression was down-regulated in LCa and it might inhibit the malignant progression of LCa through interacting with RGS17.^13^ MiR-96-5p was reported to participate in the occurrence and development of multiple tumors, including breast cancer,^14^ colon cancer,^15^ gastric adenocarcinoma,^16^ glioma,^17^ and NSCLC.^18^ GMDS-AS1 acts as a tumor suppressor gene to upregulate the expression of CYLD via sponging miR-96-5p in lung adenocarcinoma.^19^ Previous research have also found that circPTPRA suppressed Epithelial Mesenchymal Transitioning (EMT) and metastasis of NSCLC cells through sponging miR-96-5p, and the circPTPRA/miR-96-5p/RASSF8/E-cadherin axis could be leveraged as a potential treatment avenue in NSCLC.^20^

In this study, the authors analyzed the relative expression level of LDB2 and miR-96-5p in lung cancer tissues. Results showed that LDB2 was down-regulated and negatively correlated with miR-96-5p expression. The proliferation, invasion, and metastasis of H1299 cells and expressions of cycle‐associated, invasion-associated, apoptosis‐related, and proliferation-related proteins were promoted or inhibited after LDB2 knockdown or overexpression. However, miR-96-5p exerted its function by directly binding to 3′-UTR of LDB2 and regulated expression of LDB2. The miR-96-5p could promote cell proliferation, invasion, and metastasis, and regulate the protein levels of cyclinD1, MMP9, Bcl-2, Bax through ERK1/2 signaling pathway. Therefore, the authors found LDB2 was down-regulated by miRNA-96-5p and inhibited cell proliferation, invasion, and metastasis in lung cancer H1299 cells, these findings might provide a novel target for the diagnosis and treatment of lung cancer.

## Materials and methods

### Clinical samples

65 pairs of lung tumor tissues and para-carcinoma tissues involved in this study were obtained by surgical excision from Nantong tumor hospital and Nantong first people's hospital. Before surgery and in surgery, all patients received no radiotherapy or chemotherapy treatment. This investigation was approved by the Ethics Committee of the Nantong tumor hospital and Nantong first people's hospital, and informed consent was obtained from each patient before the research. The Ethics Committee study protocol number is 2020KT048. All collected tissue samples were rinsed with aseptic enzyme-free water and placed in the cryopreservation tube within 30-minutes in vitro. And then there were maintained in liquid nitrogen for subsequent experiments.

### Cell culture

Five cell lines (A549, SPAC1, H1650, H1299 and H1975) of lung cancer and one human pulmonary epithelial normal cell line (BEAS-2B) were purchased from the Chinese Academy of Sciences Cell Bank (Shanghai, China). Cells were cultured in Roswell Park Memorial Institute-1640 (RPMI-1640) (Gibco, Rockville, MD, USA) with10% fetal bovine serum (FBS) (Gibco, Rockville, MD, USA) in a 37°C, 5% CO_2_ incubator.

### Quantitative Real Time-Polymerase Chain Reaction (qRT-PCR)

Total RNAs of lung cancer tissues and cells were isolated by TRIzol (Invitrogen, Carlsbad, CA, USA). The extracted RNAs were reversely transcribed into cDNA with the PrimeScript RT reagent (TaKaRa, Kusatsu, Japan). qRT-PCR reactions were performed using SYBR Green reagent (TaKaRa, Kusatsu, Japan) by the ABI 7500 Real Time-PCR System (ABI, Foster City, CA, USA). Each experiment was repeated more than 3 times. The relative expression levels of LDB2 to β-actin and miR-96-5p to U6 were calculated by the 2^−ΔΔCT^ method, and the formula was used as follows: △△Ct =(Ct _target gene_ -C _β-actin/U6_)tumor-tissue -(Ct_target gene_-Ct_β-actin/U6_)para-cancerous tissue. The following primer sequences were used for qRT-PCR reactions: LDB2, F:5’-CGTGCGTCTACTTTGTACTGGG-3’, R:5’-TGTGGTGTGCTGGACATCTTG-3’; β-actin, F: 5’-TCAAGATCATTGCTCCTCCTGAG-3’, R:5’-ACATCTGCTGGAAGGTGGACA-3’; The Bluge-loop^TM^ miRNA qRT-PCR Primer Sets (one RT primer and a pair of qRT-PCR primers for each set) specific for miR-96-5p was designed and synthesized by Ruibo Biotechnology company (Guangzhou, China).

### Cell transfection

Negative controls (si-RNA), siRNA containing the LDB2 overexpression and interference sequence, and miR-96-5p mimics, mimics NC, inhibitor, and inhibitor NC were designed and synthesized from Ruibo Biotechnology company (Guangzhou, China). The cells were plated in 6-well plates and siRNA transfections were performed using the manufacturer's instructions of Lipofectamine 3000 (Invitrogen, Carlsbad, CA, USA). After 24 hours, the transfection efficiency was detected by qRT-PCR and western blot.

### Cell proliferation assay

5×10^3^ cells were seeded into 96-well plates. Cell proliferation was detected by CCK-8 assay (Thermo Fisher Scientific, Waltham, MA, USA) after culturing for 0h, 24h, 48h, and 72h. The Optical Density (OD) value of each well at 490 nm absorbance wavelength was measured by an enzyme-labeled instrument (Thermo Fisher Scientific, Waltham, MA, USA). Every experiment was repeated more than 3- times.

### Cell wound healing assay

Cell migration was detected by wound healing assay. The cells were seeded into 6-well plates. The 10 uL tip was used to carefully scratch the bottom of the plates. Subsequently, PBS (Gibco, Rockville, MD, USA) was used to clean twice, and the culture medium with 2.5% low serum was added at 37°C for 24h. The inverted fluorescence microscope (OLYMPUS, Tokyo, Japan) was used to take the pictures of cells, and the Image analysis software Image J was used to calculate areas of cell migration. Migration area (24h) = Black area (0h) - Black area (24h). Migration index = Migration area (24h) / Black area (0h). Every experiment was repeated more than 3-times.

### Cell transwell assay

Cell invasion was detected by transwell assay. 1×10^4^/L cells resuspended in serum-free medium were seeded into the top chamber of the insert, while medium with 20% serum was added in the lower chamber. After incubation for 24h and 48h, the chamber was taken out to fix with 4% paraformaldehyde and stained with crystal violet. The cells on the upper surface of the insert were carefully cleaned with cotton swabs. The stained cells were imaged and calculated by an inverted fluorescence microscope, and 3 fields were randomly selected. Every experiment was repeated more than 3-times.

### Luciferase reporter assay

293T cells were seeded into 24-well plates and transferred with pEZX-FR02-LDB2-3’UTR WT and pEZX-FR02-LDB2-3’UTR MUT (GenePharma Co., Ltd., China), along with miR-96-5p mimics or miR-NC following the instructions of Lipofectamine 3000 (Invitrogen, Carlsbad, CA, USA). After transfection for 48h, luciferase activity was measured by Luc-Pair™ Duo-Luciferase HS Assay Kit (GeneCopoeia, Rockville, Md, USA). Every experiment was repeated more than 3-times.

### Western blot

The total protein was extracted by Radio-Immuno-Precipitation Assay (RIPI) solution and calculated by Bicin-Choninic Acid (BCA) protein assay kit (Pierce, Rockford, IL, USA). The protein was separated by 10% Sodium Dodecyl Sulphate-Poly-Acrylamide Gel Electrophoresis (SDS-PAGE) (New Cell and Molecular Biotech, Suzhou, China) and transferred to Polyvinylidene Difluoride (PVDF) membranes (Millipore, Billerica, MA, USA). After blocking with 5% nonfat milk, the membranes were incubated with the primary antibodies at 4°C overnight and then incubated with the secondary antibodies at room temperature for 2h. The first antibodies included LDB2 (Abcam, Boston, MA, USA)cyclinD1, MMP9, Bcl-2, Bax, p-ERK1/2 and ERK1/2 (Cell Signalling Technologies, Danvers, MA, USA). Subsequently, the Tris-Buffered Saline and Tween 20 (TBST) buffer were used to wash three times. Protein bands were exposed by Enhanced Chemiluminescence (ECL) (New Cell and Molecular Biotech, Suzhou, China). Every experiment was repeated more than 3-times.

### Statistical analysis

The Statistical Product and Service Solutions (SPSS) 25.0 software and GraphPad Prism version 5.0 software were used for statistical analysis. The normal distribution data were expressed by the means ± standard error of the mean (x ± s) and the intergroup comparison was calculated by independent sample *t*-test. The non-normal distribution data were expressed by media M (X25% ∼ X75%), and the intergroup comparison was calculated by the Mann-Whitney *U* test; p < 0.05 was expected to have a significant difference.

## Results

### The expressions of LDB2 and miR-96-5p in lung cancer tissues and cell lines

**Figure 1:**
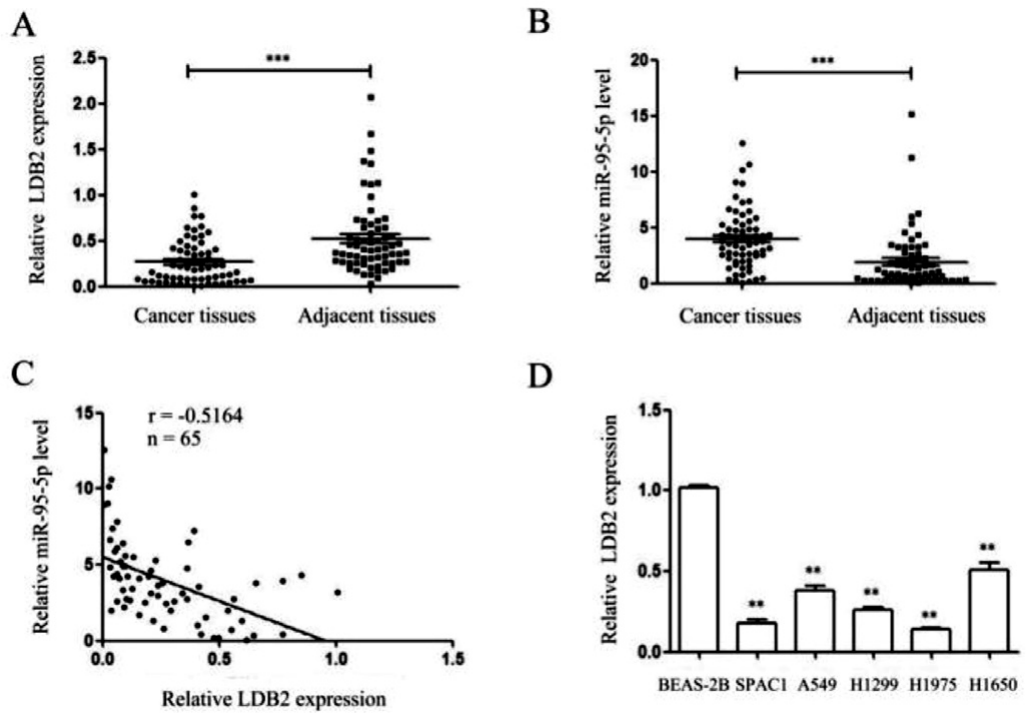
The expressions of LDB2 and miR-96-5p in lung cancer tissues and cell lines. (A‒B) qRT-PCR showed that the expression of LDB2 in lung cancer tissues was significantly lower than that in adjacent tissues, while miR-96-5p was high-regulated in lung cancer tissues; (C) The expression of LDB2 was negatively correlated with miR-96-5p; (D) The expression of LDB2 in normal bronchial epithelial cell line (BEAS-2B) and 5 lung cancer cell lines (SPAC1, A549, H1299, H1975, H1650); (** p < 0.01 and *** p < 0.001).

The authors detected the expressions of LDB2 and miR-96-5p in 65 pairs of lung cancer tissues and their corresponding para-cancerous tissues and cell lines by qRT-PCR. The present findings demonstrated that the LDB2 level in lung cancer tissues was lower than that in adjacent tissues, while miR-96-5p was high-regulated in lung cancer tissues and negatively correlated with LDB2 expression (Fig. 1A‒C). Besides, the expression of LDB2 was markedly lower in lung cancer cells than that in the normal bronchial epithelial cell line BEAS-2B (Fig. 1D). Next, the authors choose the H1299 cell which the expression was at the middle level for subsequent experiments.

### Knockdown of LDB2 promoted H1299 cell proliferation, invasion and metastasis

**Figure 2:**
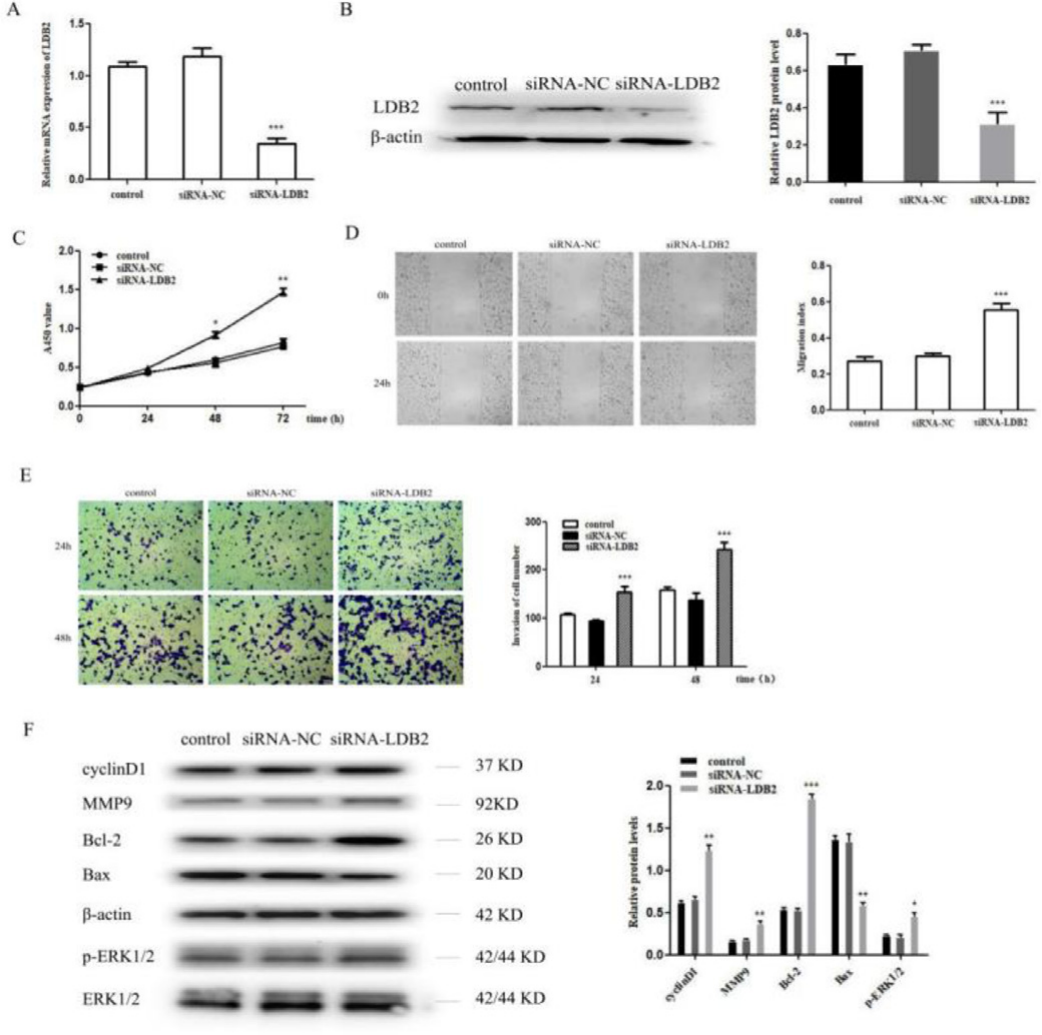
Knockdown of LDB2 promoted H1299 cell proliferation, invasion and metastasis. (A‒B) qRT-PCR and western blot revealed that LDB2 expression was significantly decreased after transfection with siRNA-LDB2 in H1299 cell; (C) CCK8 assay showed that the proliferation of H1299 cell was promoted after silencing LDB2 for 48 and 72 hours; (D) A wound healing assay demonstrated that the migration of H1299 cell was accelerated after silencing LDB2 for 24 hours; (E) A transwell assay indicated that the invasion of H1299 cell was enhanced after silencing LDB2 for 24 and 48 hours. (F) Western blotting assays for the expression of cycle‐associated, invasion-associated, apoptosis‐related and proliferation-related proteins in H1299 cells after transfection with siRNA-LDB2 (* p < 0.05, ** p < 0.01 and *** p < 0.001).

To investigate the functional roles of LDB2 in the lung cancer cell, the proliferation, invasion, and metastasis of H1299 cells were detected after transfection with siRNA-NC or siRNA-LDB2. The expression of LDB2 was significantly decreased after transfection with siRNA-LDB2 (Fig. 2A‒B), and the proliferation of H1299 cells was promoted after silencing LDB2 (Fig. 2C). Next, a wound healing assay revealed that decreased LDB2 expression accelerated migration of H1299 cell (Fig. 2D). Moreover, knockdown of LDB2 markedly promoted the invasion of H1299 cell by a transwell assay (Fig. 2E). In addition, the authors detected cycle-associated protein (cyclinD1), Matrix Metalloprotease 9 (MMP9), apoptiosis-associated proteins (Bcl-2 and Bax) and phosphorylation of Extracellular Signal-Regulated Kinase (p-ERK1/2) by western blot analysis, the results demonstrated that the protein level of cyclinD1, MMP9, Bcl-2 and p-ERK1/2 were increased while Bax was decreased in LDB2-knockdown H1299 cells (Fig. 2F).

### Overexpression of LDB2 inhibited H1299 cell proliferation, invasion and metastasis

**Figure 3:**
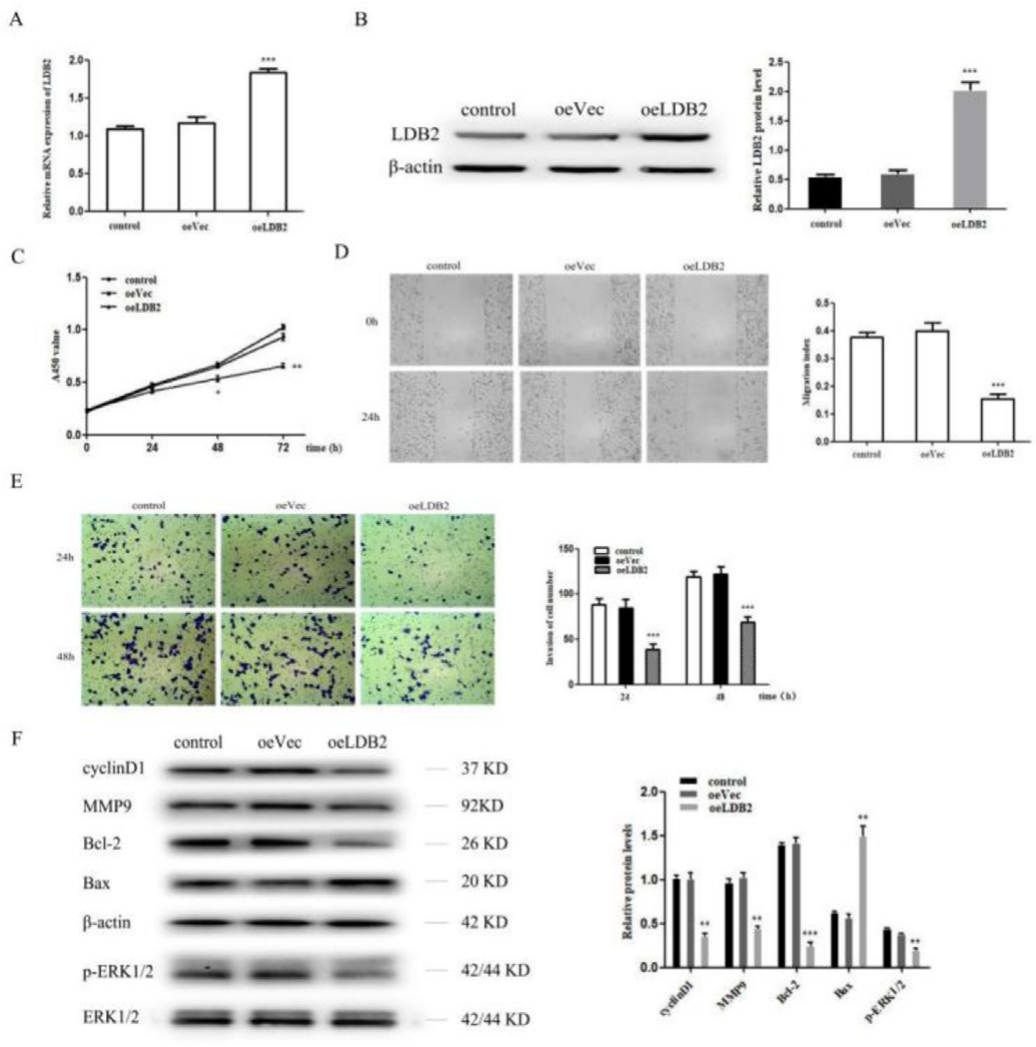
Overexpression of LDB2 inhibited H1299 cell proliferation, invasion and metastasis. (A‒B) qRT-PCR and western blot revealed that LDB2 expression was significantly increased after transfection with oeLDB2 in H1299 cells; (C) Overexpression of LDB2 inhibited H1299 cell proliferation ability as shown by CCK8 assay; (D) A wound healing assay demonstrated that overexpression of LDB2 inhibited H1299 cell migration; (E) A transwell assay indicated that LDB2 overexpression suppressed the invasion of H1299 cell. (F) Western blotting assays for the expression of cycle‐associated, invasion-associated, apoptosis‐related and proliferation-related proteins in H1299 cells after transfection with oeLDB2 (* p < 0.05, ** p < 0.01 and *** p < 0.001).

Next, the authors overexpressed LDB2 in H1299 cell (Fig. 3A‒B). LDB2 overexpression dramatically inhibited the proliferation, migration, and invasion of H1299 cells (Fig. 3C‒E). Summarily, these results indicate LDB2 plays an important role in regulating the proliferation, migration, and invasion of the lung cancer cell. Moreover, the authors also detected cyclinD1, MMP9, Bcl-2, Bax and p-ERK1/2 by western blot analysis, the results demonstrated that the protein level of cyclinD1, MMP9, Bcl-2 and p-ERK1/2 were decreased while Bax was increased in LDB2-overexpression H1299 cells (Fig. 3F).

### MiR-96-5p promoted the proliferation, invasion and metastasis of H1299 cell

**Figure 4:**
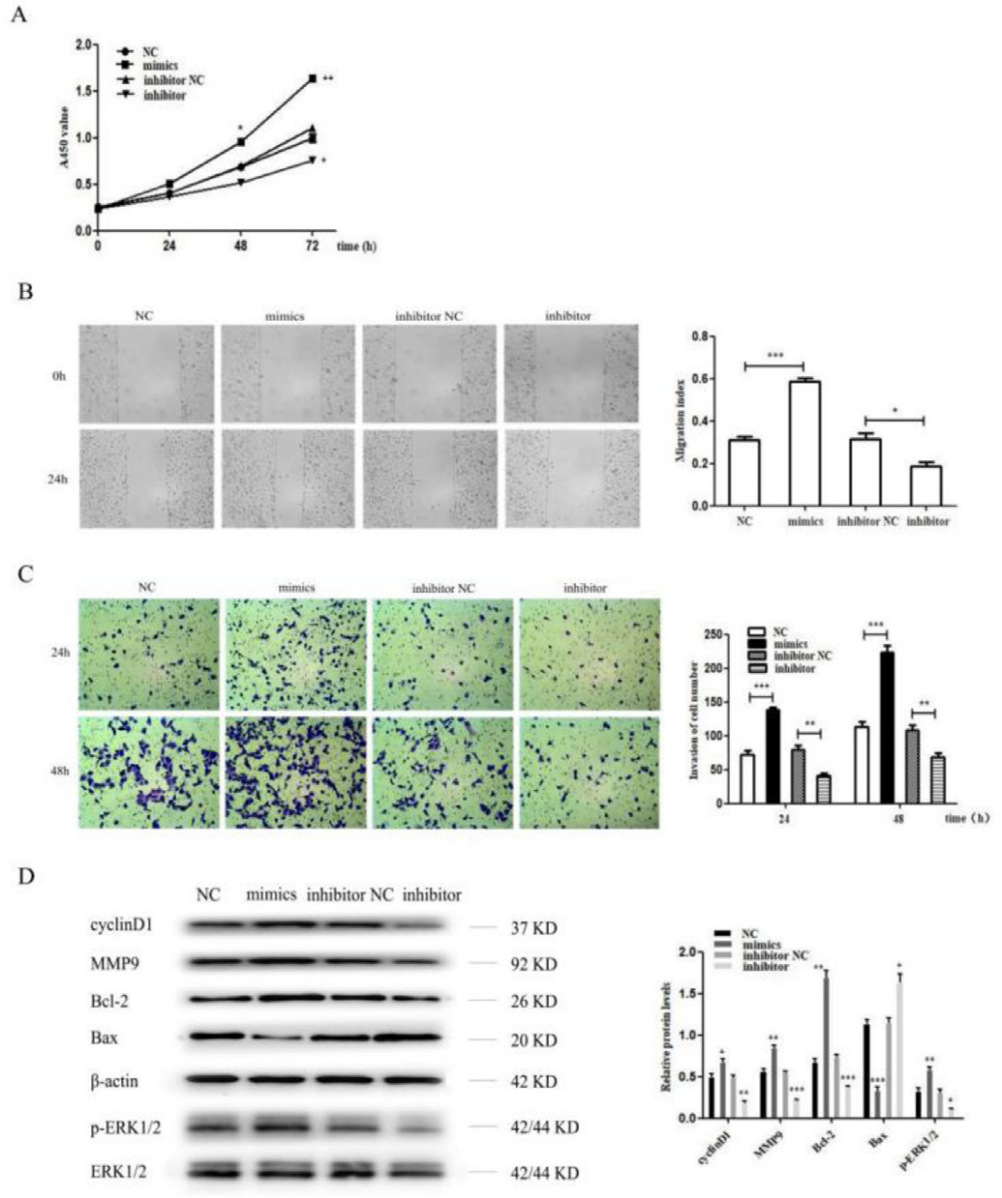
MiR-96-5p promoted the proliferation, invasion and metastasis of H1299 cell. (A) MiR-96-5p mimics promoted the proliferation of H1299 cell, while miR-96-5p inhibitor inhibited the proliferation of H1299 cell as shown by CCK8 assay; (B) A wound healing assay demonstrated that miR-96-5p mimics accelerated H1299 cell migration, while miR-96-5p inhibitor reduced H1299 cell migration; (C) A transwell assay indicated that miR-96-5p mimics enhanced the invasion of H1299 cell, while miR-96-5p inhibitor decreased the invasion of H1299 cell. (D) Western blotting assays for the expression of cycle‐associated, invasion-associated, apoptosis‐related and proliferation-related proteins in H1299 cells after transfection with miR-96-5p mimics or inhibitor (* p < 0.05, ** p < 0.01 and *** p < 0.001).

The miR-96-5p level was significantly upregulated or downregulated after transfection with the miR-96-5p mimics or inhibitor. Then, the present results suggested that the miR-96-5p mimics dramatically promoted the cell proliferation, invasion and metastasis of H1299 cell, while the miR-96-5p inhibitor suppressed the cell proliferation, invasion and metastasis of H1299 cell (Fig. 4A‒C). Moreover, the authors also detected cyclinD1, MMP9, Bcl-2, Bax and p-ERK1/2 by western blot analysis, the results demonstrated that the protein level of cyclinD1, MMP9, Bcl-2 and p-ERK1/2 were increased while Bax was decreased after transfection with miR-96-5p mimics. However, as expected, the authors obtained opposite results after transfection with miR-96-5p inhibitor (Fig. 4D).

### MiR-96-5p modulated LDB2 expression in H1299 cell

**Figure 5:**
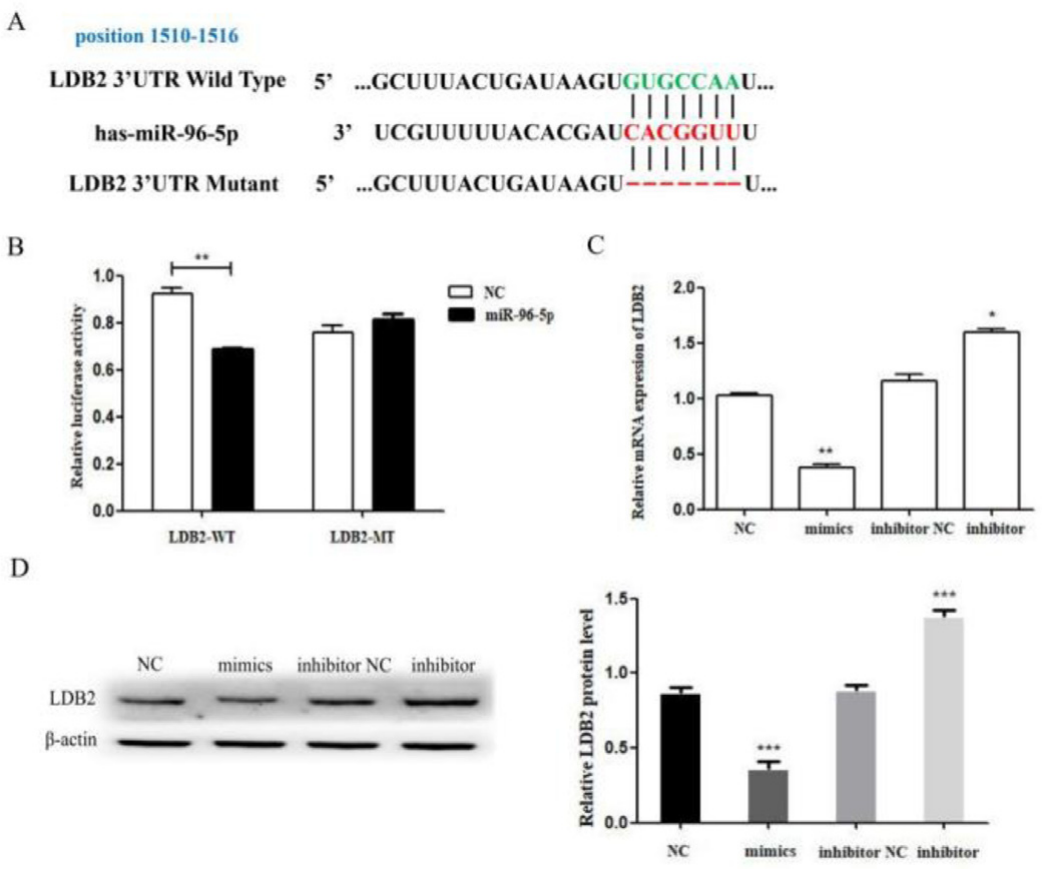
LDB2 is a target of miR-96-5p in H1299 cell. (A) The predicted binding sites of miR-96-5p in the 3’UTR of LDB2; (B) Luciferase reporter assay was used to determine the binding site; (C) The relative mRNA expression of LDB2 was decreased or increased after transfection with miR-96-5p mimics or inhibitor for 24 hours; (D) The expression of LDB2 was decreased or increased after transfection with miR-96-5p mimics or inhibitor for 48 hours (* p < 0.05 and ** p < 0.01).

According to the TargetScan and miRanda online database, the authors found a miR-96-5p binding site in the 3’UTR of LDB2 (Fig. 5A). Then, the authors found that LDB2 is a direct target of miR-96-5p by luciferase reporter assay. The results demonstrated that the luciferase activity was decreased in the wild-type group, whereas no significant difference was found in the mutant group (Fig. 5B). Next, the expression of LDB2 was detected by qRT-PCR and western blot after transfection with miR-96-5p mimics or inhibitor (Fig. 5C‒D). All these results demonstrated that LDB2 was a potential direct target of miR-96-5p in H1299 cells.

### MiR-96-5p promoted the proliferation, invasion and metastasis of H1299 cell through regulating LDB2 expression

**Figure 6:**
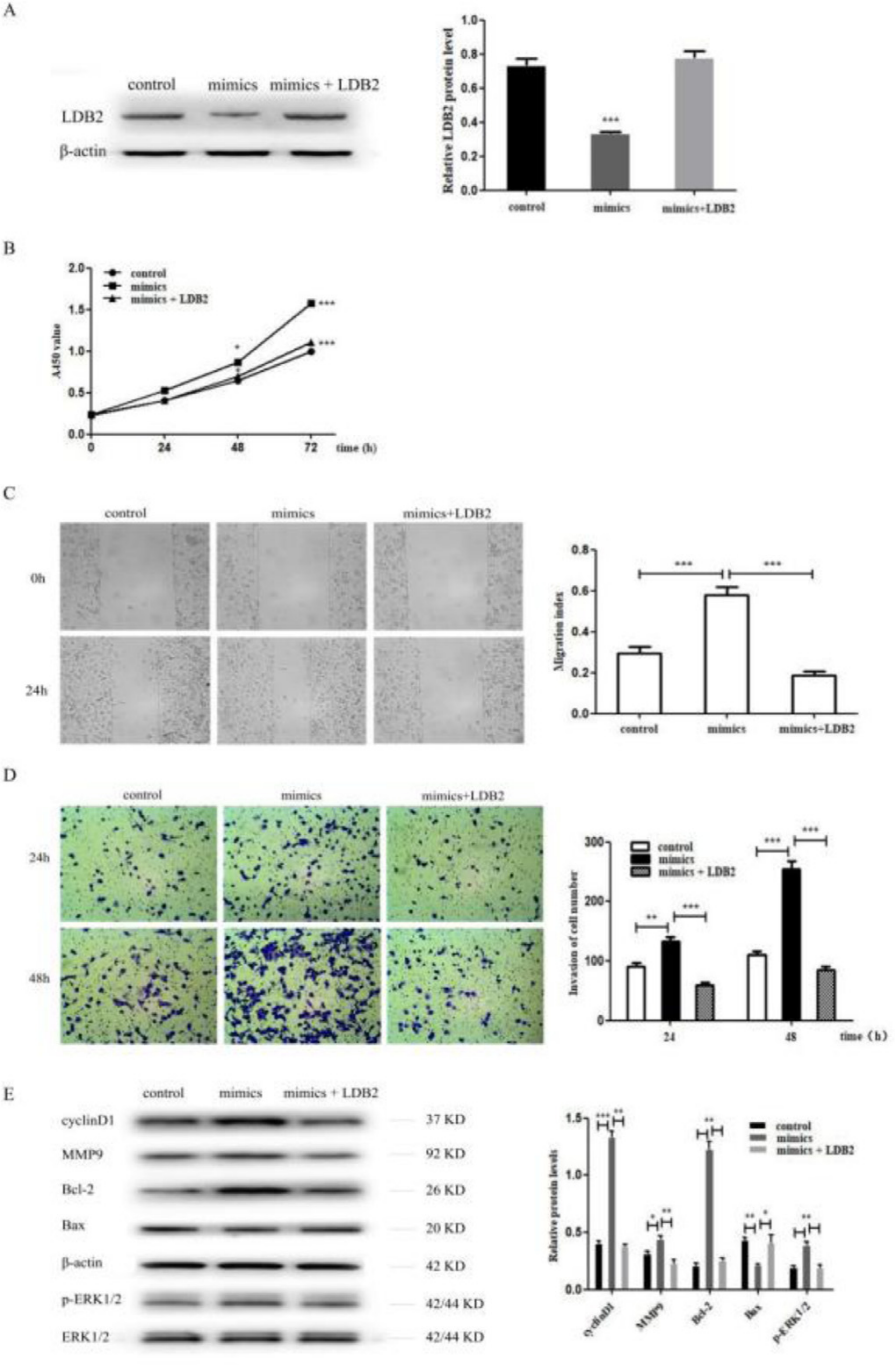
LDB2 was down-regulated by miRNA-96-5p inhibited proliferation, invasion and metastasis of H1299 cell. (A) The expression of LDB2 was detected by western blot after treating with control, mimics or mimics + LDB2 for H1299 cell; (B) The proliferation ability of control, mimics, or mimics + LDB2 treated H1299 cells was detected by CCK8 assay; (C) The migration ability of control, mimics, or mimics + LDB2 treated H1299 cells was measured by wound healing assay; (D) The invasion ability of control, mimics, or mimics + LDB2 treated H1299 cells was examined by transwell assay. (E) Western blotting assays for the expression of cycle‐associated, invasion-associated, apoptosis‐related and proliferation-related proteins after treatment with control, mimics or mimics + LDB2 for H1299 cell. (* p < 0.05 and *** p < 0.001).

To investigate whether miR-96-5p regulated the proliferation, invasion, and metastasis of H1299 cells by directly targeting LDB2, the authors co-transfected NC or miR-96-5p mimics with pcDNA 3.1+ or pcDNA-LDB2 into H1299 cell. The decreased protein expression level of LDB2 caused by the miR-96-5p mimics was dramatically restored by LDB2 overexpression (Fig. 6A). Overexpression of LDB2 abrogated the promoting effect of the miR-96-5p mimics on H1299 cell proliferation, invasion and metastasis (Fig. 6B‒D). These results validated that miR-96-5p promoted the proliferation, invasion and metastasis of H1299 cell through regulating LDB2 expression. In addition, the authors also detected cyclinD1, MMP9, Bcl-2, Bax and p-ERK1/2 by western blot analysis, the results revealed that the protein level of cyclinD1, MMP9, Bcl-2 and p-ERK1/2 were increased while Bax was decreased after transfection with miR-96-5p mimics. However, overexpression of LDB2 restored the effect of miR-96-5p mimics on protein regulation (Fig. 6E).

## Discussion

Lung cancer is one of the most common malignancies, it has the highest morbidity and mortality of any cancers. The early diagnosis and effective treatment can improve the survival rate of patients.^21^ Therefore, it is urgent to find highly sensitive and specific early markers of lung cancer. LDB2 is a highly conserved transcriptional cofactor, it has been suggested that LDB2 may be a negative regulator to inhibit the migration of mouse embryonic fibroblasts by binding Ste20-like Kinase^22^ and inhibiting the proliferation of corneal epithelial cells by promoting the expression of non-coding RNA H19.^23^ LDB2 was low-expressed in hepatocellar carcinoma, and patients with low LDB2 expression had a poor prognosis. Meanwhile, over-expression of LDB2 in hepatoma cells could significantly inhibit cell proliferation and migration, but the knockdown of LDB2 had the opposite effect. Further study showed that LDB2 could recruit the tumor suppressor protein BRD7 into the promoter of the tumor-promoting protein HEY1 and exert the anti-cancer effect.^8^ In this study, the authors found that LDB2 was under-expressed in lung cancer tissues. Knockdown or overexpression of LDB2 could promote or inhibit the proliferation of lung cancer cells, which was consistent with the results of Zhai's research.^10^ Further studies had shown that LDB2 could inhibit the invasion and migration of H1299 cells. In liver cancer cells, LDB2 inhibited cell migration and promotes cell apoptosis by up-regulating the expression of Bax and Bid and down-regulating the expression of MMP2 and MMP9.^8^ LDB2 was also found to inhibit cell proliferation by increasing the expression of cell cycle inhibitor p21.^8^^,^^23^ In addition, many studies showed that LDB2 played an important role in inhibiting angiogenesis and development. In the Chorio-Allantoic Membrane (CAM), a highly vascularized tissue, LDB2 could be significantly enriched in vascular endothelial cells and the expression of LDB2 would change with the development of blood vessels.^24^ Shang et al. found that transendothelial migration of leukocytes in LDB2-deficient mice was enhanced.^25^ LDB2 formed a transcription complex with LMO/TAL1/GATA2 and to the promoter of DLL4 to increase its expression and further suppressed sprouting and hyper-dense network formation in human umbilical vein endothelial cells.^26^ Tumor angiogenesis is a complex process involving multiple factors, steps, cells, and cytokines. Tumor cells play a key role in tumor angiogenesis. Tumor cells can secrete VEGFA, PGF, TGF-β, and other cytokines under external stimulation. The secreted cytokines can act on endothelial cell receptors and thereby affect the formation of tumor blood vessels.^27^^,^^28^ In the cornea, epithelial-specific expression of a Dominant Negative (DN) CLIM under the Keratin 14 (K14) Promoter, Gene Ontology (GO) analysis revealed that the downregulated genes were mainly enriched in focal adhesion, TGF-β, and cytokine signaling pathway.^23^ Therefore, in the angiogenesis of lung cancer, whether LDB2 directly affects angiogenesis or acts on endothelial cells through the secretion of angiogenic factors by tumor cells needs to be further researched.

In recent years, many molecular targets regulated by miRNAs have been continuously reported. more evidence suggests that miRNAs may be involved in tumorigenesis, development, and metastasis by regulating the target genes related to malignant biological behaviors, such as cell infiltration, proliferation, and apoptosis. MiR-96-5p is located in the human chromosome 7 and regulated gene expression by binding to the 3’non-coding region of target gene mRNA. MiR-96-5p is well-established as an oncogenic miRNA species in several human cancers. For example, miR-96-5p promoted breast cancer migration via activating MEK/ERK signaling.^14^ By down-regulating FOXO3 and CDKN1A, miR-96-5p could promote the proliferation of gastric cancer cells and bladder cancer cells respectively.^16^^,^^29^ Many studies have shown that miR-96-5p acting as an oncogene may play an important role in the occurrence and development of lung cancer. It was reported that miR-96-5p promoted the proliferation and migration of lung cancer cells in vitro and tumor growth and metastasis in vivo which partially depended on AIMP3-p53 axis.^30^ Also, miRNA-96-5p could target RECK,^31^ FOXO3,^18^ RASSF8^20^ and SMAD9^32^ by binding theirs 3’non-coding region and promote the malignant transformation of lung cancer. In this study, the authors found miR-96-5p was high-regulated in lung cancer tissues and negatively correlated with LDB2 expression. MiR-96-5p exerted its function by directly binding to 3′-UTR of LDB2 and regulated expression of LDB2, and it could promote the proliferation, invasion, and metastasis of H1299 cells. Moreover, the present results revealed that miR‐96-5p/LDB2 could promote cellular behavior's through

ERK1/2 pathway and regulate cell cycle regulator cyclinD1, invasion-associated MMP9, apoptosis‐related Bcl‐2 and Bax expression. All findings suggested that LDB2 was down-regulated by miRNA-96-5p inhibited cell proliferation, invasion, and metastasis in lung cancer H1299 cells, which can provide a new direction for the future diagnosis and treatment of lung cancer.

## Conclusions

In this research, the authors found that the expression of LDB2 was significantly reduced in lung cancer tissues and negatively correlated with miR-96-5p expression. MiR-96-5p may be to promote proliferation, invasion, and metastasis via targeting LDB2 and regulate cellular behaviors through the ERK1/2 signaling pathway in lung cancer.

## Authors’ contributions

Fuying Chu and Xiang Chen conceived and designed this study; Xinxin Xu, Yan Zhang and Hua Cai performed the experiments; Jingjing Peng and Hongli Liu collected important background information; Yanan Li and Han Zhang performed the statistical analysis. Fuying Chu and Xinxin Xu edited the manuscript. All authors read and approved the final manuscript.

## Conflicts of interest

The authors declare no conflicts of interest.
